# Comparison of two prognostic scores (BSI and FACED) in a Spanish cohort of adult patients with bronchiectasis and improvement of the FACED predictive capacity for exacerbations

**DOI:** 10.1371/journal.pone.0175171

**Published:** 2017-04-06

**Authors:** Edmundo Rosales-Mayor, Eva Polverino, Laura Raguer, Victoria Alcaraz, Albert Gabarrus, Otavio Ranzani, Rosario Menendez, Antoni Torres

**Affiliations:** 1 Fundació Clínic, IDIBAPS, CIBERES, Servicio de Neumología, Hospital Clinic de Barcelona, Barcelona, España; 2 Facultad de Medicina, Universidad de Barcelona, Barcelona, España; 3 CIBERES, Instituto de Investigación Sanitaria La Fe, Servicio de Neumología, Hospital Universitario y Politécnico La Fe de Valencia, Valencia, España; National and Kapodistrian University of Athens, GREECE

## Abstract

Bronchiectasis (BE) is a chronic and heterogeneous respiratory disease that requires a multidimensional scoring system to properly assess severity. The aim of this study was to compare the severity stratification by 2 validated scores (BSI and FACED) in a BE cohort and to determine their predictive capacity for exacerbations and hospitalizations. Moreover, we proposed a modified version of FACED which was created to better predict the risk of exacerbations in clinical practice. We performed a prospective cohort study including BE patients >18 years old with a follow-up period of 1-year. One-hundred eighty-two patients (40% males; mean age 68) were studied. Patients were stratified according to the number of exacerbations during the follow-up, and according to BSI and FACED scores. BSI classified most of our patients as severe 99 (54.4%) or moderate 47 (25.8%), while FACED mainly classified as mild 108 (59.3%) or moderate 61 (33.5%). BSI and FACED showed an area under ROC curve (AUC) for exacerbations of 0.808 and 0.734; and for hospitalizations (due to BE exacerbations) of 0.893 and 0.809, respectively. Subsequently, we modified FACED by adding previous exacerbations (Exa-FACED) and this new score classified patients as mild 48.4%, moderate 34.6% and severe 17.0%, with an improved AUC for exacerbations (0.760) and hospitalizations (0.820). Despite previous validations of BSI and FACED, they classified our patients very differently. As expected, FACED showed poor prognostic capacity for exacerbations. We support the Exa-FACED score to predict the risk future exacerbations for been easy to use in clinical practice.

## Introduction

Bronchiectasis (BE) is a chronic respiratory disease defined pathologically by irreversible and usually progressive dilatation and destruction of the bronchi[1]. Clinically, bronchiectasis is characterized by a persistent cough, sputum production[2], recurrent respiratory infections[3–5], progressive loss of lung function[6, 7] and worsening in quality of life[8].

BE is a multidimensional disease, so its prognosis cannot be adequately determined by one isolated variable. Identifying patients at risk of exacerbations and mortality is vital to optimize their management. Two multidimensional scoring systems have been created and validated to classify the severity of BE: the Bronchiectasis Severity Index (BSI) and the FACED scores. Both scores embrace diverse clinical, functional, radiological and microbiological aspects that are characteristics of the disease. BSI is relatively complex, evaluating 9 variables with different point values to identify patients at risk of future mortality, hospital admissions and exacerbations[9]. Alternatively, FACED is an easy-to-use system, incorporating 5 dichotomized parameters in order to classify the severity of BE according to its prognosis in terms of 5-years[10], although during its development it has not been validated for exacerbations. Exacerbations are incidental events that have an important role on treatment and patient´s quality-of-life, and associated with health-related costs. Better prediction of exacerbations may provide information to clinicians intervene in their prevention, and therefore, decrease hospitalizations and worse outcomes.

The aims of this study were to compare the classification by the 2 scores in a larger cohort of stable BE patients classified by the number of exacerbations and, investigate the predictive values of both scores in terms of exacerbations and hospitalizations due to exacerbations of BE. The FACED score was developed in a Spanish cohort[10] and is an easy-to-use score but is less consistent for predicting exacerbations[11] than BSI, accordingly we modified the FACED to classify better our patients by severity and predict exacerbations and hospitalizations.

## Materials and methods

### Design and study population

We conducted a prospective and observational study of adult patients with the previous diagnosis of bronchiectasis attending the specialized outpatient clinic of a tertiary care university hospital in Barcelona during the period 2011–2015.

We excluded patients with, a) severe immunosuppression, such as in solid-organ or bone-marrow transplantation or HIV/AIDS, or receiving chemotherapy or other immunosuppressive drugs (≥20 mg prednisone-equivalent per day for 2 weeks or more); b) active tuberculosis; c) cystic fibrosis (CF); d) pulmonary interstitial disease; and e) COPD or asthma as a primary respiratory diagnosis.

The diagnosis of bronchiectasis was confirmed by computerized tomography (CT) scan of lungs performed previous to study recruitment. Aetiology of bronchiectasis had been investigated in all patients according to Spanish guidelines[12]. Chronic bronchial infection was defined as the presence of at least 2 respiratory isolates (sputum) of the same pathogen in the previous year (3 months apart)[13].

We used the definition of bronchiectasis exacerbation of the Spanish guidelines[12]. This guideline defines an exacerbation as an acute presentation of changes in sputum (volume, consistence, purulence or haemoptysis), and/or increased dyspnoea not due to other cause. It may be accompanied by increased cough, fever, fatigue, malaise, anorexia, weight loss, pleuritic chest pain, changes in respiratory exploration (onset of abnormal breathing sounds or changes in previous ones), abnormal radiograph indicative chest infection, impaired respiratory function or increase systemic markers of inflammation. Also, it can be associated with changes in microbial density of colonizing bacteria or the acquisition of a new microorganism.

### Data collection and follow-up

Data collection included demographics, vaccinations (influenza and pneumococcal), comorbidities (Charlson Comorbidity Index), exacerbations in the previous year, chronic bronchial infection, pulmonary function test, radiological characteristics of bronchiectasis (CT scan) and aetiology, long-term antimicrobial therapy (oral antibiotic as macrolides and/or inhaled antibiotic) and concomitant medication. BSI and FACED were calculated for all patients. Follow-up was based on periodic visits every 3–6 months during 1 year.

### Outcomes

We classified patients according to the annual frequency of exacerbations at follow-up in two groups (Low-EXAC: <2/year and High-EXAC: ≥2/year). The authors chose this cut-off based in a previous analysis of our database that showed a median of 2 exacerbations at previous year. This factor is one of the major markers of disease activity and is strictly related to prognosis in the short- and long-term[7]. Therefore, our primary outcome was patients who have ≥2 exacerbations per year at follow-up (High-EXAC). We also evaluated hospitalizations due to BE exacerbations.

The study was approved by the local Ethics committee (CEIC 2013/8071) and a written informed consent was required as prerequisite for subject recruitment.

### Statistical analysis

Results were expressed as mean ± standard deviation (SD) for continuous variables and as frequency (percentage) for categorical variables. Parametric statistics were used for data confirmed to have a normal distribution using Kolmogorov-Smirnov test. Differences in continuous variables were analysed using an independent two-tailed *t*-test or Mann-Whitney *U* test, as appropriate. Categorical variables were studied using the chi-square test or Fisher exact test when necessary.

Classification of severity (BSI and FACED) was stratified into mild, moderate and severe, according to the original authors’ designations[9, 10]. The Charlson Comorbidity Index (CCI) also was calculated[14]. We used Cohen's kappa coefficient (k)[15] to measure the agreement between the scores’ severity classification. The discrimination of each score was determined by constructing a Receiver Operating Characteristic (ROC) curve and the area under the curve (AUC) and 95% confidence intervals (95% CI) were determined[16]. We compared AUC between scores using the Delong´s test. The cut-off points were calculated arbitrarily by the authors following the severity classification of the scores (mild vs. moderate/severe). Sensitivity, specificity, positive predictive value (PPV), negative predictive value (NPV), likelihood ratios (LR) and odds ratio (OR) were also calculated.

For the modification of FACED, first we identified variables among those not included in the original FACED score that were associated with exacerbations at 1 year of follow-up using univariate analysis. Second, variables with clinical rationale and statistically significant (p<0.05) were entered in a multivariate logistic regression analysis in addition to the 5 items of the original FACED score. Third, we selected the variable that remained independently associated with exacerbations after adjusting for the original FACED score variables. The Hosmer-Lemeshow goodness-of-fit test was performed to assess the calibration of the models. We report OR and 95% CI of the logistic regression models. A bootstrapping technique was used to assess the internal validation and the robustness of the multivariate logistic regression models using 1,000 samples[17]. All tests were two-tailed and significance was set at p<0.05. Data is provided in a supporting information file (S1 File).

## Results

### General characteristics of the study population

We collected data from 198 consecutive patients with diagnosis of bronchiectasis and excluded 16 patients who do not have all variables available, remaining 182 patients (92%) for analysis. The general characteristics of the population studied are show in Table 1. The most frequent aetiologies were post-infectious (47.8%). Overall, 104 (67.5%) patients had an obstructive pattern (FEV_1_/FVC<70%) and 24 (13.2%) patients had a severe airflow limitation (FEV_1_<50%).

**Table 1:** General characteristics of the study population (n = 182).

Variable	Low-EXAC (n = 139)	High-EXAC (n = 43)	p-value*	All (N = 182)
**Demographic**				
Age (years), mean ± SD	65.3 ± 14.9	76.8 ± 8.7	**<0.001**	68.0 ± 14.6
Sex (male), n (%)	55 (39.6)	15 (41.9)	0.789	73 (40.1)
BMI (kg/m2), mean ± SD	25.6 ± 4.6	25.4 ± 4.8	0.779	25.6 ± 4.6
Smoking (Current smoker or Ex-smoker), n (%)	62 (44.6)	21 (48.8)	0.626	83 (45.6)
Alcohol (Current or ex-consumer), n (%)	10 (7.7)	2 (4.7)	0.732	12 (6.9)
Influenza vaccine, n (%)	95 (68.4)	32 (74.4)	0.449	127 (69.8)
Pneumococcal vaccine, n (%)	78 (56.1)	25 (58.1)	0.815	103 (56.6)
**Comorbidities**				
Arterial hypertension, n (%)	63 (45.3)	24 (55.8)	0.229	87 (47.8)
Diabetes mellitus, n (%)	17 (12.2)	11 (25.6)	**0.034**	28 (15.4)
Cardiovascular disease, n (%)	30 (21.6)	26 (60.5)	**<0.001**	56 (30.8)
Neurological disease, n (%)	9 (6.5)	5 (11.6)	0.268	14 (7.7)
Chronic renal disease, n (%)	13 (9.4)	7 (16.3)	0.204	20 (10.9)
Chronic liver disease, n (%)	14 (10.1)	6 (13.9)	0.477	20 (10.9)
Neoplastic disease, n (%)	23 (16.6)	13 (30.2)	**0.049**	36 (19.8)
Charlson Comorbidity Index, mean ± SD	2.2 ± 1.8	3.3 ± 2.1	**0.001**	2.4 ± 1.9
**Bronchial chronic infection**				
by any microorganism (all), n (%)	31 (22.3)	19 (44.2)	**0.005**	50 (27.5)
*P*. *Aeruginosa* (PA), n (%)	20 (14.4)	18 (41.9)	**<0.001**	38 (20.9)
Other microorganism no-PA, n (%)	14 (10.1)	1 (2.3)	0.200	15 (8.2)
**Previous year exacerbations**				
Exacerbations, mean ± SD	1.5 ± 1.5	2.9 ± 2.1	**<0.001**	1.8 ± 1.8
Emergency visits, mean ± SD	0.4 ± 0.8	2.1 ± 2.1	**<0.001**	0.8 ± 1.4
Hospitalization, mean ± SD	0.2 ± 0.5	1.8 ± 1.9	**<0.001**	0.6 ± 1.2
**Spirometry**				
FEV_1_% of pred., mean ± SD	73.3 ± 21.2	54.5 ± 17.6	**<0.001**	70.3 ± 21.8
FEV_1_/FVC, mean ± SD	64.9 ± 11.5	53.8 ± 11.6	**<0.001**	63.2 ± 12.2
**Bronchiectasis’ aetiology**				
Idiopathic, n (%)	28 (20.1)	7 (16.3)	0.574	35 (19.2)
Post-infectious, n (%)	68 (48.9)	19 (44.2)	0.587	87 (47.8)
Associated to COPD, n (%)	11 (7.9)	10 (23.3)	**0.006**	21 (11.5)
Associated to Asthma, n (%)	18 (12.9)	4 (9.3)	0.604	22 (12.1)
Others, n (%)	14 (10.1)	3 (6.9)	0.766	17 (9.3)
**Dyspnoea**				
mMRC, mean ± SD	1.1 ± 1.1	1.8 ± 0.8	**<0.001**	1.3 ± 1.1
mMRC ≥2	30 (22.4)	27 (62.8)	**<0.001**	57 (32.2)
**Chest CT**				
Cystic BE, n (%)	16 (11.5)	5 (11.6)	0.983	21 (11.6)
Lobules affected with BE, mean ± SD	3.2 ± 1.7	3.2 ± 1.5	0.922	3.2 ± 1.6
**Treatment**				
Inhaled steroids, n (%)	100 (71.9)	37 (86.1)	0.061	137 (75.3)
Inhaled LAMA, n (%)	60 (43.2)	25 (58.1)	0.085	85 (46.7)
Inhaled LABA, n (%)	102 (73.4)	37 (86.1)	0.088	139 (76.4)
Oral steroids, n (%)	18 (12.9)	11 (25.6)	**0.048**	29 (15.9)
Theophylline, n (%)	2 (1.4)	2 (4.7)	0.237	4 (2.2)
Chronic inhaled antibiotic, n (%)	7 (5.0)	9 (20.9)	**0.001**	16 (8.8)
Chronic oral macrolides, n (%)	24 (17.3)	12 (27.9)	0.126	36 (19.8)
**Severity Score**				
FACED, mean ± SD	1.8 ± 1.5	3.2 ± 1.7	**<0.001**	2.1 ± 1.7
FACED (mild / moderate / severe), %	68 / 28 / 4	33 / 51 / 16	**<0.001**	59 / 34 / 7
FACED (moderate + severe), n (%)	45 (32.4)	29 (67.4)	**<0.001**	74 (40.7)
BSI, mean ± SD	7.9 ± 4.6	13.2 ± 4.0	**<0.001**	9.2 ± 4.9
BSI (mild / moderate / severe), %	25 / 32 / 43	5 / 5 / 90	**<0.001**	20 / 26 / 54
BSI (moderate + severe), n (%)	105 (75.5)	41 (95.4)	**0.004**	146 (80.2)

Percentages calculated on non-missing data.

* Chi-square test, Fisher’s exact test, Student´s t-test or Mann-Whitney U test, as appropriate.

Abbreviations: SD = Standard deviation. COPD = Chronic obstructive pulmonary disease. FEV1 = Forced expiratory volume in the first second. FVC = Forced vital capacity. PA = *Pseudomonas aeruginosa*. CT = Computed tomography. BMI = Body mass index (kg/m^2^). MRC = Medical Research Council dyspnoea scale (score 1 to 5). mMRC = modified MRC scale (score 0 to 4). BE = bronchiectasis. LAMA = long-acting muscarinic antagonist. LABA = long-acting beta-agonist.

Chronic bronchial infection was observed in 50 patients (27.5%); *Pseudomonas aeruginosa* (PA) being the most frequent 38 (20.9%) followed by *Haemophilus influenzae* 13 (7.1%), *Moraxella catarrhalis* 3 (1.6%) and *Staphylococcus aureus* 3 (1.6%).

Overall, in the previous year 47 patients (25.8%) had no exacerbations, 45 (24.7%) suffered one exacerbation and 90 (49.5%) had 2 or more exacerbations.

During the prospective follow-up of 1 year, 32 patients (17.6%) had only 1 exacerbation, 43 (23.6) had 2 or more exacerbations, and 107 (58.8%) had no exacerbations. Of patients with at least one exacerbation during follow-up, 65% (49 of 75) required hospitalization. Therefore, we separated our patients in two groups as was described in methods: Low-EXAC 139 patients (76.4%) and High-EXAC 43 (23.6%).

FACED classified our patients mainly as of mild or moderate severity, with few patients as severe; while BSI scored most patients as severe (Table 2). Table 2A shows the classification by both scores and agreement rates. There was a slight concordance between two scores, BSI *versus* FACED (k = 0.17).

**Table 2:** Classification of patients by BSI versus FACED (2A) or Exa-FACED (2B) scores, and FACED versus Exa-FACED (2C) scores. (Total N = 182)

**2A.**				
**BSI**	**FACED**			**Agreement**
	**Mild**	**Moderate**	**Severe**	
**Mild**	36	0	0	Kappa = 0.166 (p<0.001). Concordance 36.8%
**Moderate**	29	18	0	
**Severe**	43	43	13	
**2B.**				
**BSI**	**Exa-FACED**			**Agreement coefficient**
	**Mild**	**Moderate**	**Severe**	
**Mild**	34	2	0	Kappa = 0.269 (p<0.001). Concordance 47.3%
**Moderate**	26	21	0	
**Severe**	28	40	31	
**2C.**				
**FACED**	**Exa-FACED**			**Agreement coefficient**
	**Mild**	**Moderate**	**Severe**	
**Mild**	88	20	0	Kappa = 0.643 (p<0.001). Concordance 79.1%
**Moderate**	0	43	18	
**Severe**	0	0	13	

Data are expressed as n.

### Comparison of low-EXAC and high-EXAC patients

High-EXAC patients were older, had high prevalence of PA chronic bronchial infection, worse CCI score, FEV_1_, FEV_1_/FVC, and mMRC scores, and increased use of oral steroids and chronic inhaled antibiotic, compared with Low-EXAC patients. BE associated to COPD was more frequent in High-EXAC patients. No differences were observed in radiological pattern of bronchiectasis at CT and chronic use of inhaled corticosteroids and bronchodilators (Table 1).

### Comparison of BSI and FACED in Low-EXAC and High-EXAC patients

We compared the frequency of individual components of FACED and BSI scores between Low-EXAC and High-EXAC patients (Table 3). For FACED, we found statistically significant differences for the chronic bronchial infection by PA and age components between Low-EXAC and High-EXAC. For BSI, we found significant differences between groups in chronic bronchial infection by PA and age components (as in FACED) and of 2 other components that are not included in the FACED score: prior hospital admission and prior exacerbations.

**Table 3:** Comparison of BSI and FACED according to their components (n = 182).

Score		Component (points)	Low-EXAC (N = 139)	High-EXAC (N = 43)	p-value*
**FACED**					
**FEV**_**1**_**%**		≥50% (0 point)	124 (89.2)	34 (79.1)	0.086
		<50% (2 points)	15 (10.8)	9 (20.9)	
**Age**		≤70 years (0 point)	79 (56.8)	10 (23.3)	**<0.001**
		>70 years (2 points)	60 (43.2)	33 (76.7)	
**Chronic PA**		No (0 point)	119 (85.6)	25 (58.1)	**<0.001**
		Yes (1 point)	20 (14.4)	18 (41.9)	
**CT extension**		1–2 lobes (0 points)	61 (43.9)	16 (37.2)	0.439
		>2 lobes (1 points)	78 (56.1)	27 (62.8)	
**Dyspnoea**		mMRC 0–2 (0 points)	133 (95.7)	35 (81.4)	**0.002**
		mMRC 3–4 (1 points)	6 (4.3)	8 (18.6)	
**BSI**					
**Age**		<50 (0 point)	25 (17.9)	0 (0)	**<0.001**
		50–69 (2 point)	50 (35.9)	10 (23.2)	
		70–79 (4 point)	36 (25.9)	14 (32.6)	
		≥80 (6 point)	28 (20.2)	19 (44.2)	
**BMI**		<18.5 (2 point)	134 (96.4)	41 (95.4)	0.669
		≥18.5 (0 point)	5 (3.6)	2 (4.6)	
**FEV**_**1**_**%**		>80% (0 point)	57 (41.0)	21 (48.9)	0.058
		50–80% (1 point)	67 (48.2)	13 (30.2)	
		30–49% (2 points)	13 (9.4)	6 (13.9)	
		<30% (3 points)	2 (1.4)	3 (7.0)	
**Prior hospital admission**		No (0 points)	67 (48.2)	4 (9.3)	**<0.001**
		Yes (5 points)	72 (51.8)	39 (90.7)	
**Prior exacerbations**		0–2 (0 points)	110 (79.1)	20 (46.5)	**<0.001**
		≥3 (2 points)	29 (20.9)	23 (53.5)	
**MRC**		1–3 (0 points)	133 (95.7)	35 (81.4)	**0.002**
		4 (2 points)	4 (2.9)	8 (18.6)	
		5 (3 points)	2 (1.4)	0 (0)	
**PA colonization**		No (0 points)	119 (85.6)	25 (58.1)	**<0.001**
		Yes (3 points)	20 (14.4)	18 (41.9)	
**No PA colonization**		No (0 points)	125 (89.9)	42 (97.7)	0.200
		Yes (1 points)	14 (10.1)	1 (2.3)	
**Radiological severity**		No (0 point)	60 (43.2)	15 (34.9)	0.335
		>3 lobes or cystic BE (1 point)	79 (56.8)	28 (65.1)	

Data are expressed as n (%).

* Chi-square test or Fisher’s exact test, as appropriate.

Abbreviations: FEV1 = Forced expiratory volume in the first second. PA = *Pseudomonas aeruginosa*. CT = Computed tomography. BMI = Body mass index (kg/m^2^). MRC = Medical Research Council dyspnoea scale (score 1 to 5). mMRC = modified MRC scale (score 0 to 4). BE = bronchiectasis.

### Predictive performance evaluations

**Fig 1:**
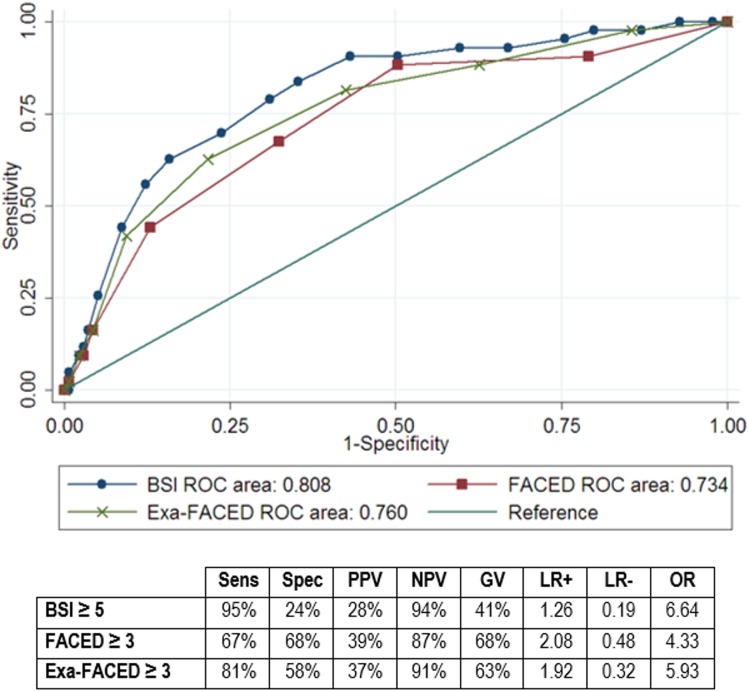
ROC curve for ≥2 exacerbations at 1-year of follow-up. Abbreviations: Sens = sensitivity; Spec = specificity; PPV = positive predictive value; NPV = negative predictive value; GV: global value; LR+ = positive likelihood ratio; LR- = negative likehood ratio; OR = odds ratio; NA = non-available.

**Fig 2:**
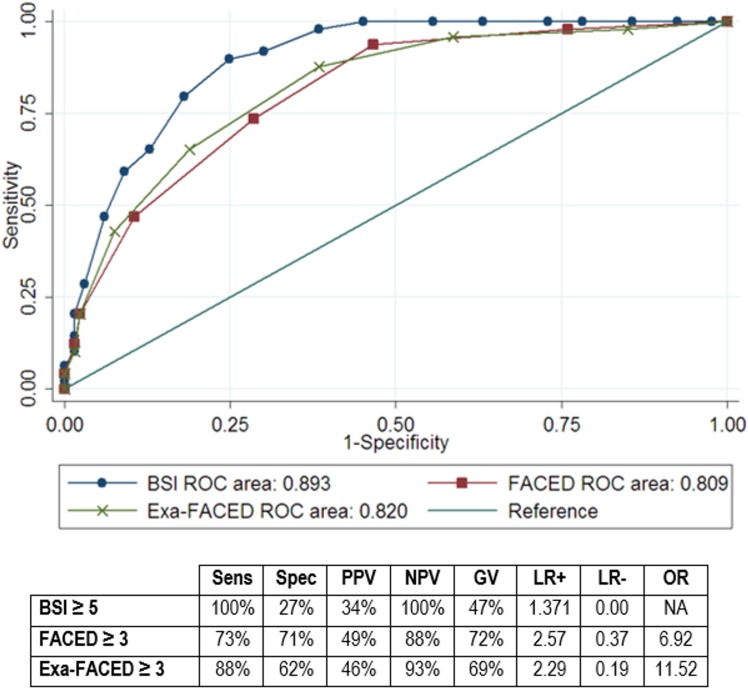
ROC curve for ≥1 hospitalizations at 1-year of follow-up. Abbreviations: Sens = sensitivity; Spec = specificity; PPV = positive predictive value; NPV = negative predictive value; GV: global value; LR+ = positive likelihood ratio; LR- = negative likelihood ratio; OR = odds ratio; NA = non-available.

The discrimination of each score to predict exacerbations (≥2/year) or hospitalizations (≥1/year) are shown in Figs 1 and 2. The AUC was 0.808 (95%CI 0.734–0.882) for BSI and 0.734 (95%CI 0.648–0.821) for FACED regarding exacerbations (p = 0.023 for BSI *vs* FACED). We observed a higher sensitivity of BSI in contrast with a higher specificity of FACED using FACED≥3 and BSI≥5 as cut-off points (moderate and severe classes).

Regarding hospitalizations, the AUC was 0.893 (95%CI 0.848–0.938) for BSI and 0.809 (95%CI 0.744–0.875) for FACED (p<0.001 for BSI *vs* FACED). Again, BSI showed higher sensitivity but lower specificity than FACED.

### Modified version of FACED (Exa-FACED)

Table 1 shows that comorbidities (diabetes, cardiovascular and neoplastic diseases), exacerbations history, BE associated to COPD and usual treatment (oral steroids and chronic inhaled antibiotic) are more frequent in the HIGH-EXAC patients, apart from other variables already included in the BSI or FACED score. The variables chosen for being statistically significant variables, with clinical relevance and not included in the original FACED score, are reported in Table 4. After multivariate adjustment, the only variable which remained independently associated with exacerbations was the history of 2 or more exacerbations in the last year (Table 4). Additionally, as previous hospitalizations are associated with exacerbations, we performed another multivariate analysis model (data not show) changing the independent variable of exacerbations for the variable “history of 1 or more hospitalization due an exacerbation last year”, and, as we seen previously, only this variable (hospitalization) was predictive for exacerbations at follow-up independently of the 5 items of the FACED (OR 4.69; 95%CI 1.88–11.69; p = 001). The internal validation using bootstrapping demonstrated robust results with small 95% CI around the original coefficients.

**Table 4:** Univariate and multivariate analysis of variables associated with the risk of 2 or more exacerbations.

Univariate analysis				Multivariate analysis (*)			
Variables	OR	95% CI	p-value	OR	95% CI	p-value	β-Coefficient
CCI ≥ 3	2.39	1.19–4.80	**0.014**	1.46	0.64–3.31	0.365	0.378
**Exacerbation ≥ 2/last year**	4.06	1.89–8.72	**<0.001**	2.57	1.07–6.15	**0.034**	**0.944**
BE associated to COPD	3.53	1.38–9.01	**0.008**	2.44	0.79–7.54	0.120	0.894
Oral steroids	2.31	0.99–5.38	0.052	-	-	-	-
Chronic inhaled antibiotic	4.99	1.73–14.37	**0.003**	2.29	0.62–8.45	0.214	0.829

* The 5 items of the original FACED score (Pseudomonas aeruginosa chronic infection, dyspnoea by mMRC score, forced expiratory volume in the first second, age in years, and number of lobes affected with bronchiectasis at the CT) were additionally included in the logistic regression model. Hosmer and Lemeshow goodness-of-fit test: p = 0.666.

Therefore, we decided to modified the FACED incorporating a new item for “exacerbations” (considering positive the presence of ≥2 exacerbations or ≥1 hospitalization due an exacerbation last year) to the original FACED score (the **Exa-FACED**). After rounding the β-coefficient for the new variable, the presence of this item assigned 1 point to the total score (Table 5), which punctuates from 0 to 8 (instead of 0–7 of the original score).

**Table 5:** Exa-FACED score.

Severity marker		Points
*Pseudomonas aeruginosa* chronic infection		
	No	0
	Yes	1
Dyspnoea mMRC score		
	0-II	0
	III-IV	1
FEV_1_		
	≥ 50% of predicted	0
	< 50% of predicted	2
Age		
	< 70 years	0
	≥ 70 years	2
Number of lobes involved		
	1–2	0
	> 2	1
≥ 2 exacerbations and/or ≥ 1 hospitalizations due to an exacerbation (*)		
	No	0
	Yes	1

An overall score from 0 to 8 points is derived as a sum of the scores for each variable. Severity classification: mild (0–2), moderate (3–4), severe (≥5).

* Additional item

Because of the short period of follow-up (1 year), we did not evaluate the prediction of mortality as for the original FACED score, but decided to use the same severity stratification of FACED: mild 0–2 points, moderate 3–4 points or severe ≥ 5 points.

We reclassified our cohort with the new score and we observed a new distribution which is intermediate between FACED and BSI particularly for mild and severe classes (the percentage of moderate patients remains stable in all scores). In fact, Exa-FACED classifies patients as mild in 48.4% (FACED 59.3%; BSI 19.8%), moderate in 34.6% (FACED 33.5%; BSI 25.8%) and severe in 17.0% (FACED 7.2%; BSI 54.4%). The concordance between Exa-FACED and BSI was fair (k = 0.27) but higher than that observed between FACED and BSI (Table 2).

A ROC curve was also constructed for Exa-FACED and sensitivity and specificity was calculated considering positive moderate and severe classes (Exa-FACED≥3) as cut-off point. For the risk of ≥2 exacerbations/year (Fig 1), the AUC was 0.760 showing a similar sensitivity than BSI, with and intermediate specificity between BSI and FACED. There was a significant difference in AUC of Exa-FACED with FACED (p = 0.039) but no with BSI (p = 0.114). For the risk of ≥1 hospitalizations/year (Fig 2) the AUC was 0.820 and it proved to have a high sensitivity with an intermediate specificity. There was a significant difference in AUC with BSI (p = 0.001) but no with FACED (p = 0.347).

## Discussion

The main findings of this study were the following:

1. There were some differences between patients with low or high frequency of exacerbations at follow-up.
2. Both scores (BSI and FACED) classified our patients very differently and there was a poor concordance between the two scores.
3. BSI had a better predictive capacity than FACED for the occurrence of exacerbations or hospitalization due to an exacerbation at follow-up.
4. The inclusion of a new item to the original FACED, “Exacerbations”, improved the predictive capacity of this score to be almost similar to BSI but with less complexity to fill it. We called this modified version “Exa-FACED”.

A correct stratification by severity is fundamental in order to improve the clinical management of patients, allowing appropriate preventive and therapeutic interventions and also to facilitate research in the field in order to properly analyse and interpret research data and to identify patients for clinical trials.

BSI and FACED were validated by large observational single-centre and multicentre studies[9–11, 18]. In our cohort of BE patients, with a higher proportion of PA chronic respiratory infection (21%, consistent with the data described in the literature[19, 20]), we observed substantial differences in the severity stratification by both scores and a poor concordance between (Table 2). As expected, the severity stratification by FACED and BSI showed relevant differences in the patient distribution by severity. FACED scored most patients as mild and BSI scored most as severe.

Minov et al.[21] achieved a similar distribution of disease severity by BSI and FACED, but the study showed some underlying limitations, principally a small sample size (n = 37) and a low proportion of patients with PA chronic bronchial infection (8%). On the contrary, in a single-centre cohort of 91 patients with the same rate of chronic PA (22%)[18] and in a multicentre study[11] of 1612 patients, similar classification differences were found in the severity assessment, as we observed. Also, a proportion of patients had a discordant classification; Ellis et al.[18] scored severe in BSI but mild in FACED in 11 subjects out of 74 (maximal discordance in 15% of the population), and we scored the same in 43 subjects out of 182 (maximal discordance in 24%).

### Causes of different severity stratification by FACED and BSI

In order to investigate the differences between the two scores we compared the prevalence of individual items composing the scores in our 2 groups of BE patients. We found significant difference in 5 variables out of 9 in the BSI score: age, dyspnoea, chronic infection by PA and the presence of prior exacerbations and hospitalizations (see Table 3). We therefore believe that the main causes of the observed differences are the different punctuation of PA chronic bronchial infection, which has been related to an increase in the risk of exacerbations and mortality in BE, and the presence of exacerbations and hospitalizations[7, 22–25] which are not considered in FACED.

If we analyse the burden of these significantly different variables (PA, exacerbations, hospitalizations) for each score, we observe that they contribute more or exclusively to BSI than to FACED, resulting in a higher punctuation and a tendency to classify patients as moderate or severe with BSI. Merely by having a hospital admission due to exacerbation in the previous 2 years, BSI automatically classifies the patient as moderate (5 points out of 5) and contributes to more than 50% (5 points out of 9) to the severe class, while this parameter is not taken into consideration with FACED. Similarly, suffering ≥3 exacerbations provides 40% of the punctuation for moderate (2 points out of 5) and more than 20% for the severe class (2 points out of 9). This variable is neither considered in FACED.

Secondly, the contribution of chronic bronchial infection by PA to the severity punctuation is superior in BSI than in FACED, both in the moderate (BSI 60% [3 points out of 5]; FACED 33% [1 point out of 3]) and the severe (BSI 33% [3 points out of 9]; FACED 20% [1 point out of 5]) class. Finally, we also found a greater contribution of other variables to the BSI than to the FACED punctuation, although some of them were not significantly different between the 2 groups: age (for all the divisions 50–69, 70–79 and ≥80 years), low BMI and chronic bronchial infection by other microorganisms (non-PA).

### Ability to predict exacerbations and hospitalizations

Our results show that neither BSI nor FACED score are excellent systems to predict exacerbations or hospitalizations in BE patients. However, in our cohort BSI had a superior ability to predict exacerbations compared with FACED, and especially with those exacerbations that require hospitalization. Nevertheless, BSI also shows a lower specificity than FACED, probably due to the fact that it considers numerous different items in the scoring system.

### Exa-FACED, a modified version of FACED

Since none of the scores showed an excellent ability to predict exacerbations and hospitalizations, and considering the differences observed between both scores, we decided to modify the FACED score. This score is potentially easy to use in clinical practice compared to BSI (5 dichotomized variables in FACED vs. 9 variables with different point values in BSI) but it is not validated to predict exacerbations[10]. With this modified version, we intend to increase the predictive capacity of FACED for exacerbations and hospitalizations and to maintain its specificity (superior in FACED than in BSI) and ease of use.

Actually, with the new Exa-FACED we achieved an intermediate classification of our patients by severity, scoring for mild, moderate and severe in percentages between BSI and FACED. This more balanced distribution might have a better association with disease activity and severity. Besides, the correlation between Exa-FACED and BSI is better than the correlation between the original FACED and BSI but it still remains weak. Also, Exa-FACED proved to predict the risk of exacerbations almost like BSI and more than FACED. However, Exa-FACED demonstrated to predict hospitalizations less then BSI but similarly to FACED score.

Since FACED score was already validated for mortality risk, we are sufficiently confident with the fact that Exa-FACED should maintain the same prognostic value in terms of mortality, becoming overall an ideal prognostic score of both exacerbations and mortality in clinical practice due to its accompanying ease of use.

### Potential limitations and strengths of the study

The current study has some limitations. First, it is a single-centre study with a relatively small number of patients, which could have certain influence on the obtained data and their interpretation. Secondly, it does not include a long-term follow-up after 1 year, lacking the ability to prove mortality prediction of the different scores. Although we did an internal validation with bootstrapping technique, the Exa-FACED will require an external validation by further international studies including larger populations.

Among the strengths of the present study, it is important to notice the broad characterization of our patients, including a deep analysis of the aetiology and exacerbations, because of a follow-up in a specialized centre by professionals with experience in bronchiectasis. Moreover, our study is proposing a new and better tool for assessment of severity and prognosis in BE (Exa-FACED) that may easily be applied to clinical practice and dedicated to research in the field.

## Conclusions

As we expected, BSI and FACED classify our patients very differently. BSI showed a superior ability to predict exacerbations and hospitalizations compared with FACED. Furthermore, we demonstrated that a modified version of FACED score incorporating the variable “exacerbations” and/or “hospitalizations” better classifies severity and risk of future exacerbations and hospitalizations in our cohort of patients. We support the use of Exa-FACED for clinical practice due to its ability to predict exacerbations, hospitalizations and mortality (the latter as widely demonstrated for FACED[10]) and its ease of use.

## Supporting information

S1 File Minimal dataset. (DTA)
